# Parameter Identification and Validation of Shape-Memory Polymers within the Framework of Finite Strain Viscoelasticity

**DOI:** 10.3390/ma14082049

**Published:** 2021-04-19

**Authors:** Ehsan Ghobadi, Alexey Shutov, Holger Steeb

**Affiliations:** 1 Institute of Applied Mechanics (CE), University of Stuttgart, Pfaffenwaldring 7, 70569 Stuttgart, Germany; ehsan.ghobadi@gmail.com; 2 Lavrentyev Institute of Hydrodynamics, Lavrentyeva 15, 630090 Novosibirsk, Russia; alexey.v.shutov@gmail.com; 3 Stuttgart Center for Simulation Science, University of Stuttgart, Pfaffenwaldring 5a, 70569 Stuttgart, Germany

**Keywords:** finite viscoelasticity, parameter identification, modeling, functional materials, shape memory effect, shape memory polymer, physical aging, swelling

## Abstract

Shape-Memory Polymers (SMPs) can be stretched to large deformations and recover induced strains when exposed to an appropriate stimulus, such as heat. This emerging class of functional polymers has attracted much interest and found applications in medicine and engineering. Nevertheless, prior to any application, their physical and mechanical properties must be thoroughly studied and understood in order to make predictions or to design structures thereof. In this contribution, the viscoelastic behavior of a polyether-based polyurethane (Estane) and its rate- and temperature-dependent behavior have been studied experimentally and by the mean of simulations. The model-inherent material parameters are identified with the assumption of the thermo-rheological complexity. Here, the numerical results of uni-axial stress relaxations were compared with the associated experiments in conjucation with the Levenberg-Marquard optimization method to determine the parameters of the Prony equation. The ability of the model to simulate the thermo-mechanical properties of Estane was evaluated by data-rich experimental observations on tension and torsion in various temperature ranges. Heterogeneous tests are included into the experimental program to cover a broader spectrum of loading scenarios.

## 1. Introduction

Understanding the thermochemical and thermomechanical coupling states in materials is very important and necessary from various points of view, especially for designing smart materials and estimating the durability of various industrial products. This research area investigates the interaction of mechanical and chemical forces in metals and polymers, as well as temperature dependent effects.

Among the various industrial materials, polymers are more sensitive to such mechanical and chemical forces. This is due to their chemical structure and also their morphology. Amorphous and semi-crystalline polymers can be easily filled with low molecular weight compounds, such as water and other chemical solvents, when they come into contact with them. This would lead to morphology changes and structural defects, as the polymer chains would then be pulled apart by small molecules diffused into them. As a result, their characteristic temperatures, such as the glass transition temperature ($\theta_{g}$) or crystallization temperature ($\theta_{c}$), will change and their effective functional temperature range varies clearly.

One of the most sensitive materials to even small thermochemical and thermomechanical coupling states are Shape-Memory-Polymers (SMPs). Shape-Memory Effect (SME) is an ability of a SMP to be deformed and manipulated to a fixed temporary shape until an appropriate trigger is utilized for transformation of temporary shape to a memorized original shape [1,2,3]. There are a variety of shape storage and triggering mechanisms for different polymer systems [4,5]. However, polymers with appropriate chemical structure and morphology should be programmed and processed prior to triggering with, e.g., extrusion, electro-spinning, or 3-D printing, in order to achieve the Shape-Memory (SM) capability [6]. The SME appears normally due to heating and deforming of the system above its transformation temperature ($\theta_{\mathrm{sw}}$), which could be glass transition ($\theta_{g}$) or meting temperature ($\theta_{m}$) and subsequent cooling while the deformation is kept constant for solidification of chain segments and shape-fixation. Next, this deformed SMP can be exposed to a temperature above its $\theta_{\mathrm{sw}}$ to transform its temporary shape to permanent shape. This cycle is called Shape-Memory Creation Cycle (SMC) and could be repeated several times [7,8]. It should be noted that any diffusion of low molecular weight compounds into the polymer matrix would upset the thermochemical and thermomechanical coupling states in the materials and lead to SMC failure.

Thermoplastic phase-segregated multi-block copolymers, like our investigated polyether urethane Estane (Lubrizol, Ovele Westerlo, Belgium), are very interesting materials because of their mechanical stability and the capability of showing SME. Moreover, their permanent shape can be easily achieved through common processing approaches. However, computational modeling studies and predictive models are needed to design and optimize their SM-properties prior to any technical application. In this content, constitutive relations between the field variables, e.g., stress (σ), strain or stretch (ε or λ), and temperature (θ), are of interest.

Elastic and hyperelastic behavior of SMPs can be studied by simple (static) tensile testing, whereby their viscoelastic properties should be studied, e.g., by relaxation- or creep experiments. Both of these properties can also be described by mathematical models. As an example, the hyperelastic behavior of polymeric samples can be explained by strain-invariant-based models, like the one of Mooney-Rivlin [9] or Yeoh [10], or by principle-stretched-based models, like that of Ogden [11]. Marckmann et al. [12] proposed a thorough comparison of twenty hyperelastic models for rubberlike materials and analyzed their abilities to reproduce different types of loading conditions.

As a matter of course, choosing a correct mathematical model and precise determination of material parameters is very crucial as it has a significant impact on the accuracy and reliability of the results. The usual way to find the parameters of a model is as follows: a series of proper and relevant experiments is performed and then by mathematical describing the physical behavior in the test, the results of the experiments are fitted to the mathematical model. Here, normally, simple methods, like stochastic techniques and evolution strategies, optimization procedures for inherent parameter identification based on the Nedler-Mead simplex algorithms, and other procedures, are employed [13,14,15]. Although no gradient information is needed in all these methods their performance is poor. Therefore, sometimes multi-axial tests are performed employing complex sample geometries and resulting inhomogeneities, which also have their own disadvantages since the inverse calculations are computationally demanding [16,17,18].

Twizell et al. [19] used the optimization algorithm method of Levenberg-Marquardt and determined the material constants of the Ogden model. Saleeb et al. [20] introduced an issue of developing effective and robust schemes to implement a class of the Ogden type hyperelastic constitutive models, for large strain analysis of rubber-like materials.

Constitutive relations for viscoelastic materials can be obtained from elastic and viscous elements. In order to elucidate the viscoelastic and viscoplastic responses of polymers, two hypotheses have been used: (I) the split of the free energy of the viscoelastic solid into an equilibrium and non-equilibrium part and (II) the multiplicative decomposition of the deformation gradient into an elastic and viscous part [21]. In this framework, Huber et al. [22], proposed a rheological three-parameter model to describe the mechanical behavior of materials in a limited range of small deformations and extended it to large strains.

Park and Schapery [23] used the Prony series to describe the relaxation and creep behavior of a viscoelastic material. Diebels et al. [24] identified the elastic and viscoelastic material parameters from constitutive equations by means of a Tikhonov regularization and inspired an extra penalty term from the stress-strain relationships to expect better results. Haupt et al. [25] used a relatively simple identification method based on the concept of fractional calculus and obtain the model-inherent material parameters. Yoshida et al. [26] suggested a constitutive model comprised of two elastoplastic and hyperelastic parts. The elastoplastic part includes a strain-dependent isotropic hardening law and the hyperelastic part incorporates the damage model. Amin et al. [27] introduced a hyperviscoelastic model to explain the mechanical behavior of rubbers. Their model consists of a nonlinear viscous coefficient to represent the rate dependent behavior and is validated through relevant tests for compression and shear regimes.

The second method for determination of material constants involves the use of Finite Element Methods (FEM). Some researchers simulated the experimental investigations with Finite Element Analysis and so identified the material parameters. This method has the advantage that (I) the complexitiy of heterogeneous problems are more in consideration than in homogeneous analytic investigations, and (II) the obtained parameters are often more accurate. As an example, Petera et al. used Finite Element Analysis for improvement of viscometry results obtained by a cone-plate rheometer [28]. Ghoreishy [29] has also benefited from Finite Element Method and determined the parameters of the Prony series in a hyperviscoelastic material model.

Aside from the above mentioned methods, Huang et al. [30] used nanoindentation tests to measure the complex moduli of linearly viscoelastic materials through an indentation process with a spherical indenter. Beake [31] has also utilized this novel testing technique to investigate the creep behavior of thin semi-crystalline and amorphous polymers. Experimental data were adapted to a logarithmic equation relating the fractional increase in penetration depth during creep and predicted the extension and creep ratio for different maximum loads. The influence of temperature on viscoelastic behavior of SMPs is especially important. A polymer system is said to be thermo-rheological simple if all relaxation times are affected by temperature in the same way. Thus, by application of the Time-Temperature Superposition Principle (TTSP) and the Williams-Landel-Ferry (WLF) equations, it is possible to emerge mastercurves using a reduced time variables or shift factors to obtain a broader time (frequency) domain for the data of the system [32]. In previous contributions, we have performed frequency sweep tests under torsion and determined storage and loss shear moduli mastercurves and computed the Prony constants for the tested material as solutions of a minimization problem for Tikhonov functionals [33,34]. Pacheco et al. [35] proposed a methodology for characterization of material parameters of thermo-rheologically and piezorheologically simple systems and determined the Prony series based on a mixed optimization technique of Genetic Algorithms and Nonlinear Programming.

Nevertheless, based on our comprehensive experimental investigations [34], it is now clear that considering Estane as a thermo-rheological simple material is a bad assumption. We have shown that this presupposition leads to inadequate results. Therefore, a new approach should be established to represent the temperature dependence of the viscoelastic properties [36]. In the following contribution, we show that based on the presumption of thermo-rheological complexity and finding the right material parameters through uni-axial relaxation tests for finite strains, the functional properties can be very well simulated. Here, the experimentally observed effects are exhibited by a finite viscoelastic and incompressible material model and enhanced by new approach of temperature-dependency. The material parameters are strategically identified by Levenberg-Marquardt algorithm, and results are validated through stress relaxation experiments under torsion.

## 2. Materials and Methods

**Figure 1:**
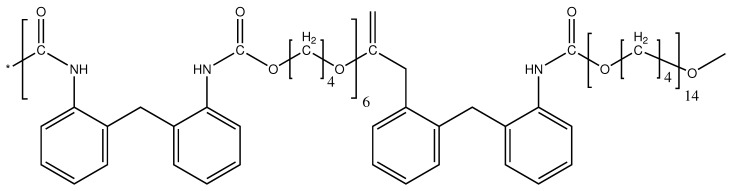
The chemical structure of Shape-Memory polyetherurethane Estane.

In the following essay, a polyether-based thermoplastic polyurethane, commercially available under the name of Estane (Lubrizol, Ovele Westerlo, Belgium), is used without further purification. Estane is a block-copolymer, synthesized from Methylendiphenylisocyanate (MDI) and 1,4- Butanediol with a polyether. The chemical structure of each reacting components is depicted in Figure 1. For Estane, a number average molecular weight of about 132 kg mol${}^{-1}$ was reported using gel permeation chromatography (GPC) [8].

For sample preparation, Estane granules are processed using an injection molding machine (Arburg Allrounder 270M 500–210, Lossburg, Germany) with an injection temperature of about 204 ${}^{\circ }$C and the outer temperature of the injection barrel of about 30 ${}^{\circ }$C. Furthermore, the samples are molded with an injection rate of 26 mm${}^{-1}$, an injection pressure of 60 MPa and a holding pressure of 55 MPa for 15 s. After processing, the plates were kept in a vacuum desiccator to keep them dry. At the end, prior to quasi-static experiments and Dynamic Mechanical Thermal Analysis (DMTA), the samples are punched either to rectangular samples or dumbbell-shaped specimens, using a manual knuckle joint press.

### 2.1. Dynamic Mechanical Thermal Analysis

**Figure 2:**
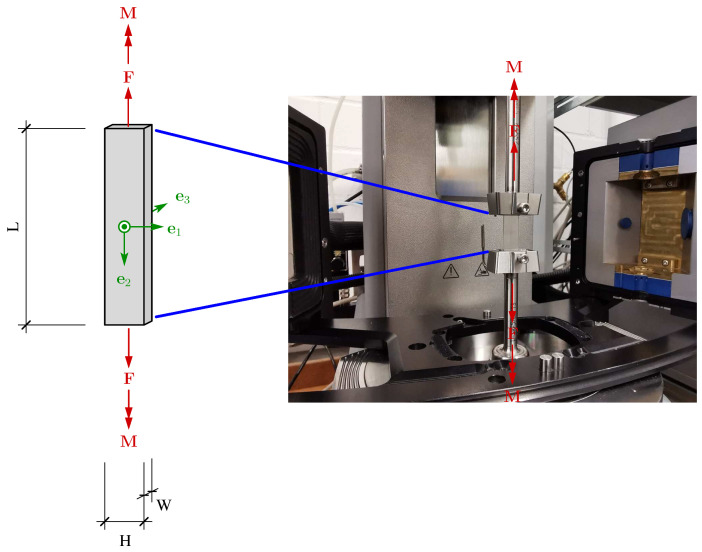
Experimental set-up for torsional loading (torque M) and investigated rectangular SM sample for thermo-rheological experiments.

Using rectangular samples with dimensions of W × H × L: 2 mm × 10 mm × 50 mm (Figure 2), DMTA experiments were accomplished in torsion mode with a torque-controlled rheometer and integrated Peltier-based temperature chamber (Anton Paar Physica MCR 702 Twin Drive plus CTD 180, Graz, Austria). A small uni-axial tensile force of around 0.5 N is applied to maintain the specimen under net tension. Thereupon, with a constant heating rate of 0.25 ${}^{\circ }$C min${}^{-1}$, temperature sweep tests with prescribed amplitude (0.01%) and different constant frequencies ranging from 0.5 to 16 Hz were performed. Here, the temperatures in the range of −20 to 120 ${}^{\circ }$C could be adjusted and kept constant with a precision of ±1 °C. Such experiments provide important insights into the effective viscoelastic properties of the investigated material. For frequency sweep tests, samples have been tensioned as before under isothermal conditions in a frequency range of [0.1–100] Hz. Figure 2 demonstrates schematically the investigated rectangular sample under torsional load as performed here. In an attempt of adequately characterize the temperature and the time dependency of the material response, transient stress relaxation experiments were performed. To conduct such quasi-static experiments, the dumbbell-shaped specimens of the type DIN EN ISO 527-2 and dimensions of W × H × L: 2 × 4 × 75 mm${}^{3}$ (25 mm parallel sample length) were pneumatically fixed along their stretching axis on the rig in displacement-driven control mode.

To calibrate the displacement of the applied testing device with the local strains of the samples at ambient conditions (out of the environmental chamber), an own custom-made optical strain measurement system has been used. This leads to a one-to-one calibration of local strains and global displacements of the test rig. To do so, we performed our experiments on vertically positioned screw-driven electromechanical test frame Schenk-Trebel RM 50 operated in displacement-driven control mode. Digital control of the test rig and data acquisition was performed using the acquisition system DOLI EDC580 (DOLI Elektronik GmbH, Münsingen, Germany) which is interfaced to the host software DOLI “Test & Motion” (version 3.0) using a proprietary networking protocol. In particular, a properly calibrated S-Type load cell was used during this work, namely, a large strain-gouge-type load cell with a maximum application range of up to 500 N. The samples are fixed along their longitudinal axis, where the bottom end is fixed stationary and the upper end is moved during deformation. The motion of the upper end will be referred to as the machine displacement *U* (mm). The load cell was attached to the upper clamp. Pneumatic grips with a grip pressure of 5 bar are used to clamp the samples. The grips allow for a force-controlled clamping. The grip faces are serrated and incorporated fences such that the samples can be positioned at the same location and relative motion between sample and grip face during deformation would be minimized. By using this custom-made real-time optical extensometer, the principal stretches and the corresponding principal strains in longitudinal and transversal directions are quantified. This optical strain measurement is a tool chain of image processing programs. Explicitly, a Charge-Coupled Device (CCD)-type industrial camera with resolution of 1024 × 768 pixel is used to acquire gray-scale raster-based images of the polymer sample during experiments. Mounted on a stepper-motor-driven mechanical stage, the camera is moved with half of the velocity and in the same direction of the machine traverse such that the relative motions between the center of the sample and the camera are minimized. Prior to that, a circle-shaped mark with a radius of r = 1.5 mm was painted on the center of the sample using laser-cut stencils and proper printing marker. The printing color is chosen such that the contrast between sample color and marking paint is as high as possible after gray-scale conversion. In this particular case, black color was used on our transparent Estane. As the sample is stretched, an initially circle-shaped mark will undergo a continuous affine transformation in accordance to the motion of the samples’ geometry, such that, for any spatial configuration, the set of points on the boundary of the mark, i.e., pixel data points, will satisfy the canonical ellipse equation

(1) $$\frac{{\left(x-x_{t,0}\right)}^{2}}{a_{t}^{2}}+\frac{{\left(y-y_{t,0}\right)}^{2}}{b_{t}^{2}}=1,$$

with ($x_{t,0},y_{t,0}$) denoting the center of the ellipse.

Then, in the absence of rotation, by determining the ellipse’s major and minor axes ($a_{t}$ and $b_{t}$, respectively), which now coincide with the Cartesian axes, the principal stretches and strains could be determined. Here, an optimization of least-square type using Equation (1) and the pixel data points of the boundary of ellipse is performed to compute the parameters of interests.

Samples have been heated to the experimental temperature with a heating rate of 3 ${}^{\circ }$C min${}^{-1}$. Once the temperature has been achieved, the specimens were equilibrated for 20 min and then deformed with a speed of 25 mm min${}^{-1}$ to a local maximum stretch of λ=2. This maximum stretch was kept constant for a holding time (relaxation time) of $t_{\mathrm{hold}}\;$= 90 min. The decay of stress over time was then investigated at different temperatures ranging from 10 to 70 ${}^{\circ }$C. Assuming a linear viscoelastic behavior and as long as no viscous flow is presented, the stress will ultimately decrease to an equilibrium stress S${}_{\mathrm{eq}}$, following multiple superimposed relaxation processes. To determine material parameters for further simulations, these experiments have been used explicitly.

### 2.2. Torsional Relaxation Experiments

There is no single experimental procedure which would permit to follow the viscoelastic behavior of a polymer over the whole range of time and temperature. In the present work, validation of identified material parameters and numerics considering theoretical background from continuum mechanics is performed by stress relaxation experiments under torsion.

To do so, rectangular samples with dimensions W × H × L: 2 mm × 4 mm × 50 mm were bounded to the upper and lower clamps of the rheometer, respectively, and equilibrated for 20 min at different experimental temperatures: $\theta_{\exp}\;=45,60,75,90$ and 105 ${}^{\circ }$C. Once the experimental temperature was reached, the samples were twisted up to a twist angle of $=360^{\circ }$ with the twist rate of $\dot{\varphi }=3.6$°/s. The torsional relaxation was then performed by fixing the twist angle at isothermal conditions up to 2 h.

### 2.3. Thermal Expansion Coefficient Experiments

Complementary to DMTA and quasi-static experiments in uni-axial or torsional modes, the thermal behavior of Estane and in particular its Coefficient of Thermal Expansions (CTE) have been studied by thermal mechanical Analysis (TMA). The CTE was determined by Thermal Mechanical Analyzer (TMA) Metler-Toledo TMA/SDTA 841 (Wien, Austria). A 2-mm thick sample was placed between two thin quartz disks and positioned on the TMA holder. Afterwards, a ball-point probe of thickness of 3 mm was positioned on the top of the sample to ensure a uniform distribution of the exerted force over the entire probe surface. Then, a very small net force of about 0.02 N was implemented to maintain a good contact between the sample and probe surface without deforming it. Finally, the probe was three times heated from 5 to 140 ${}^{\circ }$C and cooled down subsequently, whereby the third run was used for data validation. It should be noted that the both first runs were performed to eliminate any relaxation effects or eigenstresses of the specimen.

### 2.4. Finite Strain Maxwell-Zener Model

First, let us consider a specific version of the finite strain Maxwell-Zener model, which is an essential part of the constitutive model used in this study. Although a great amount of different formulations has been proposed for the Maxwell-Zener model, the preference is given to the multiplicative approach, due to numerous advantages (cf. Reference [37]). The multiplicative Maxwell model is covered as a special case by the viscoplasticity model presented by Simo and Miehe [38]. Interestingly, the Simo and Miehe model can be obtained in a number of different ways, using constitutive assumptions, which may seem unrelated [39,40,41,42]. In the current study, we employ the standard Lagrangian version of the Simo and Miehe model. We follow the presentation given by Lion [43]. For simplicity, thermostatic conditions are assumed here, where the temperature is assumed given. A thermodynamically consistent generalization to a fully coupled thermo-mechanical state can be carried out [44]. Let F(x,t) be the deformation gradient mapping a line element dX of the reference configuration to the line element dx of the current configuration. We start with the multiplicative split of the deformation gradient F into an elastic part ${\hat{F}}_{e}$ and the inelastic part $F_{i}$

(2) $$F={\hat{F}}_{e}\cdot F_{i}.$$

In the context of large strain viscoelasticity, this split is known as the Sidoroff decomposition [45]. Next, we consider the right Cauchy-Green tensor C and its inelastic counterpart $C_{i}$, both operating on the reference configuration:

(3) $$C:=F^{T}\cdot F\;\mathrm{and}\;C_{i}:=F_{i}^{T}\cdot F_{i}.$$

The Helmholtz free energy per unit mass is assumed to be of neo-Hookean type [46]

(4) $$\psi =\psi \left(C\cdot {C_{i}}^{-1}\right)=\frac{\mu }{2\;\rho_{R}}\left(\mathrm{tr}\left[\bar{C\;{C_{i}}^{-1}}\right]-3\right),\;\bar{A}:={\left(\det\left(A\right)\right)}^{-1/3}\;A,$$

where μ stands for the shear modulus, $\rho_{R}$ is the mass density in the reference configuration, tr(·) is the trace operator, and $\bar{\left(\cdot \right)}$ denotes the unimodular part of a tensor. According to the Coleman-Noll procedure, the 2nd Piola-Kirchhoff stress tensor $\tilde{S}$ is computed through

(5) $$\tilde{S}=2\;\rho_{R}\frac{\partial \psi (C\cdot {C_{i}}^{-1})}{\partial C}{|}_{C_{i}=\mathrm{const}}=\mu \;C^{-1}\cdot {\left(\bar{C}\cdot C_{i}^{-1}\right)}^{D}.$$

The evolution equation for $C_{i}$ takes the form

(6) $${\dot{C}}_{i}=\frac{1}{\eta }{\left(C\cdot \tilde{S}\right)}^{D}\cdot C_{i}=\frac{\mu }{\eta }{\left(\bar{C}\cdot C_{i}^{-1}\right)}^{D}\cdot C_{i}=\frac{2}{\tau }{\left(\bar{C}\cdot C_{i}^{-1}\right)}^{D}\cdot C_{i},$$

where η stands for viscosity, and τ=2η/μ is the inherent relaxation time. The initial conditions at time instance $t^{0}$ are specified as

(7) $$C_{i}{|}_{t=t^{0}}=C_{i}^{0}.$$

The model Equations (5) and (6) are objective, thermodynamically consistent, and w-invariant (For a general definition of the w-invariance the reader is referred to Reference [47]). The model exhibits a fading memory behavior. More precisely, the exact solution is exponentially stable with respect to small perturbations of the initial data [48]. The exact solution exhibits the following important geometrical property

(8) $$C_{i}\left(t\right)\in M\;\mathrm{if}\;C_{i}^{0}\in M,$$

where the manifold M constitutes the set of symmetric unimodular tensors

$$M:=\left\{A\in Sym:\det A=1\right\}.$$

In the following modifications, the relaxation time may depend on the temperature: τ=τ(θ). It follows from (8) that $C_{i}$ remains positive definite if $C_{i}^{0}>0$.

### 2.5. Generalized Viscoelasticity

**Figure 3:**
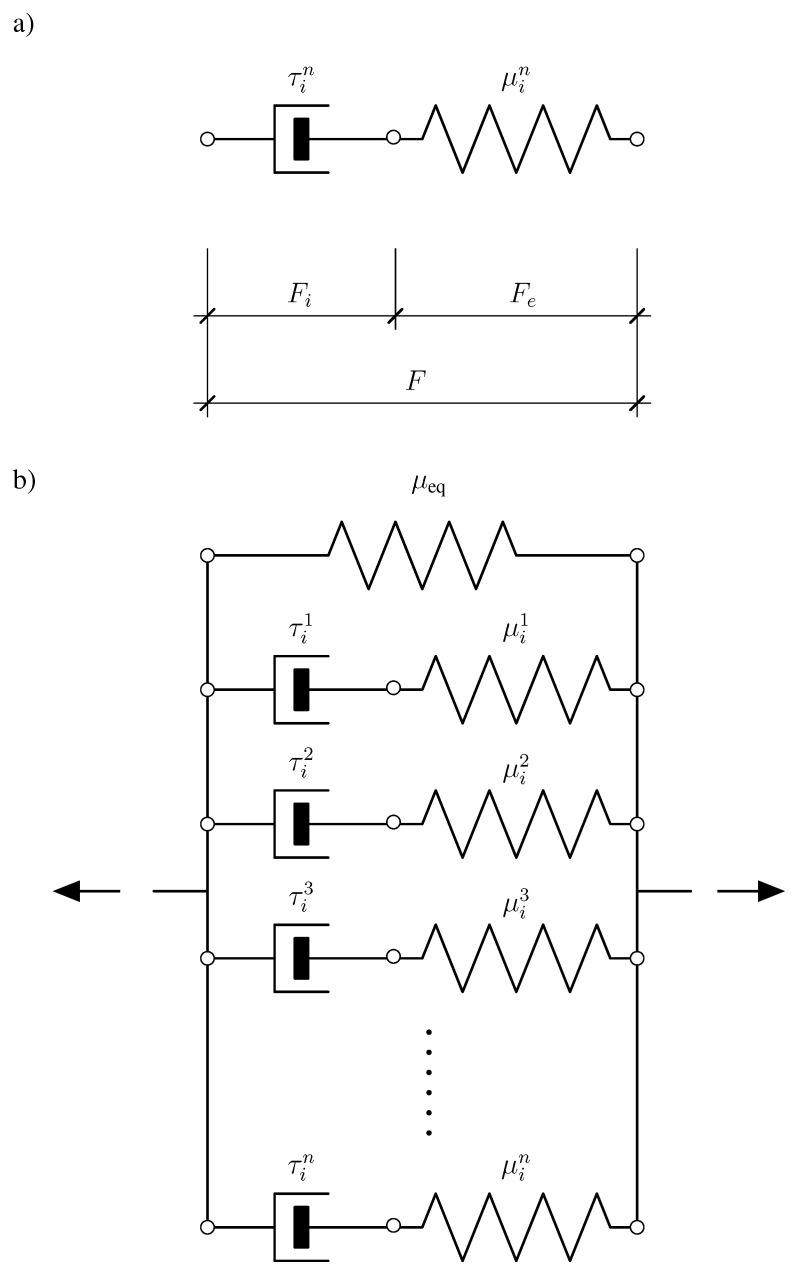
(**a**) Rheological model of the Maxwell body and (**b**) Representation of the rheological model for a generalized Maxwell (Maxwell-Zener) body with *n* Maxwell branches connected in parallel for description of the viscoelastic properties of Estane.

Now, we proceed to a model of generalized viscoelasticity, known as generalized Maxwell model (also known as Wiechert or Maxwell-Zener model) [49]. The corresponding rheological interpretation contains a spring element (Hookean body) and *n* Maxwell bodies; see Figure 3b. All the Maxwell bodies are connected in parallel. The Hookean body is used to represent the equilibrium stresses and the Maxwell bodies are introduced to capture viscous effects. The total free energy is given by a sum of isotropic functions (cf. Reference [50])

(9) $$\psi =\psi_{\mathrm{eq}}\left(C\right)+\sum_{m=1}^{n}\psi_{\mathrm{ov},m}\left(C\cdot C_{i,m}^{-1}\right).$$

Here, $\psi_{\mathrm{eq}}$ is the equilibrated part of the free energy stored in the spring element and $\psi_{\mathrm{ov},m}$ is the energy of the *m*th Maxwell body, which corresponds to the non-equilibrated part. The equilibrium spring is modeled by the neo-Hookean ansatz reinforced by a volumetric contribution (cf. Reference [51])

(10) $$\rho_{R}\psi_{\mathrm{eq}}\left(C\right)=\frac{\mu_{\mathrm{eq}}}{2\;\rho_{R}}\left(\mathrm{tr}\bar{C}-3\right)+\frac{k}{50}\left({\left(\det C\right)}^{5/2}+{\left(\det C\right)}^{-5/2}-2\right).$$

Here, $\mu_{\mathrm{eq}}$ represents the shear modulus of the material in the equilibrium state and *k* stands for the bulk modulus. Each of the Maxwell bodies is modeled by Equations (5) and (6) presented in the previous subsection. Thus, the Helmholtz free energy for the $m^{th}$ Maxwell body is given by a potential of the neo-Hookean type

$$\rho_{R}\psi_{\mathrm{ov},m}=\rho_{R}\psi_{\mathrm{ov},m}\left(C\cdot C_{i,k}^{-1}\right)=\frac{\mu_{m}}{2}\left(\mathrm{tr}\left[\bar{C\cdot C_{i,m}^{-1}}\right]-3\right),\;m=1,2,\ldots ,n.$$

Here, $\mu_{m}\geq 0$ is the shear modulus of the $m^{th}$ element and $C_{i,m}$ is the corresponding inelastic tensor of right Cauchy-Green type. The evolution of each of these variables is governed by equations of type (6), which takes the form

(11) $${\dot{C}}_{i,m}=\frac{2}{\tau_{m}}{\left(\bar{C}\cdot C_{i,m}^{-1}\right)}^{D}\cdot C_{i,m},\;C_{i,m}{|}_{t=t^{0}}=C_{i,m}^{0},$$

where $\tau_{m}\geq 0$ is a material parameter related to the relaxation time, and $C_{i,m}^{0}$ is the initial value.

Equation (9) implies that the overall second Piola-Kirchhoff stress $\tilde{S}$ is given by the sum

(12) $$\tilde{S}={\tilde{S}}_{\mathrm{eq}}+\sum_{m=1}^{n}{\tilde{S}}_{\mathrm{ov},m},\;\mathrm{where}$$

(13) $${\tilde{S}}_{\mathrm{eq}}=\mu_{\mathrm{eq}}\left\{C^{-1}\cdot {\bar{C}}^{D}\right\}+\frac{k}{10}\left\{{\left(\det\;C\right)}^{5/2}-{\left(\det\;C\right)}^{-5/2}\right\}\cdot C^{-1},$$

(14) $${\tilde{S}}_{\mathrm{ov},m}=\mu_{m}\;C^{-1}\cdot {\left(\bar{C}\cdot C_{i,m}^{-1}\right)}^{D},\;m=1,2,\ldots ,n.$$

The generalized viscoelasticity model inherits some properties of the Simo and Miehe formulation of the Maxwell model, discussed in the previous subsection. In particular, it is objective and thermodynamically consistent; it also exhibits fading memory. The exact solution of the evolution equations exhibits the geometric properties of type (8): $C_{i,m}\in M$ for all m=1,2,…,n. In general, the relaxation times $\tau_{m}$ depend on temperature θ. To explain this dependency, one should assume the system as either thermo-rheological simple or complex. As explained before, in the case of thermo-rheologically simple materials, the Time-Temperature Superposition Principle (TTSP) can be used (see Section 3.2):

(15) $$\tau_{m}=a_{T}^{j}\tau_{m}^{\left(r\right)},\;\;m=1,2,\ldots ,n,$$

where $\tau_{m}^{\left(r\right)}$ are the relaxation times at the reference temperature $\theta_{\mathrm{ref}}$.

As will be shown in the following, modeling of Estane as a thermo-rheological complex material leads to much better results. The specific dependency of relaxation times upon temperatures will be discussed in Section 3.2.1.

### 2.6. Numerical Implementation

For simplicity, we consider here only the processes with a prescribed temperature. By $\tau_{m}$, denote the corresponding relaxation times. Let us consider a typical time step $t_{n}\mapsto t_{n+1}$, $\Delta t:=t_{n+1}-t_{n}>0$. Assume that the right Cauchy-Green tensor at $t_{n+1}$ equals ${}^{n+1}C$. Within the context of a displacement-based finite element method, ${}^{n+1}C$ is known at each point of the Gauss integration. Let the previous values of the inelastic Cauchy-Green tensor for different Maxwell branches be equal to ${}^{n}C_{i,m}$. Our goal is to integrate the evolution Equation (11) within the time step. Since, in many applications, the time step size Δt may be very large compared to some of the relaxation times $\tau_{m}$, an implicit time stepping is needed. For the Simo-Miehe version of the Maxwell model with the energy storage of neo-Hookean type, an explicit update formula was presented in Reference [52]. In our notation, it reads

(16) $${}^{n+1}C_{i,m}=\bar{{}^{n}C_{i,m}+2\frac{\Delta t}{\tau_{m}}\;\bar{{}^{n+1}C}},\;m=1,2,\ldots ,n.$$

Although this formula corresponds to an implicit discretization, *it is iteration free*. It can be used for large time steps, if needed. In particular, ${}^{n+1}C_{i,m}\to \bar{{}^{n+1}C}$ as $\frac{\Delta t}{\tau_{m}}\to \infty$. A non-iterational generalization of this formula to cover the energy storage of the Mooney-Rivlin type is presented in Reference [53].

The corresponding numerical scheme is first order accurate; it exactly preserves the geometric property ${}^{n+1}C_{i,m}\in M$ even for very large time steps. The exact preservation of the incompressibility, in turn, suppresses the accumulation of the numerical error [48]. Since ${}^{n+1}C$ is known, the evolution equations for the different Maxwell branches are integrated separately. After the current values ${}^{n+1}C_{i,m}$ have been found, the overall stress is computed according to (12)–(14). The presented algorithm is implemented into the commercial finite element code MSC.MARC, employing the user subroutine HYPELA2. Obviously, any alternative forms of the equilibrium elastic potential $\psi_{\mathrm{eq}}$ can be implemented without essential changes of the presented procedure.

## 3. Results

### 3.1. Investigation of the Influence of Temperature on Rheological Properties

#### 3.1.1. DMTA—Temperature Sweep Tests

As previously emphasized, the viscoelastic properties of a material which are time and temperature dependent can be distinguished from thermo-rheological experiments. The depiction of significant changes in morphology of a polymer can be highlighted as its feature and, thus, well suitable for analysis of rheological properties of polymers.

**Figure 4:**
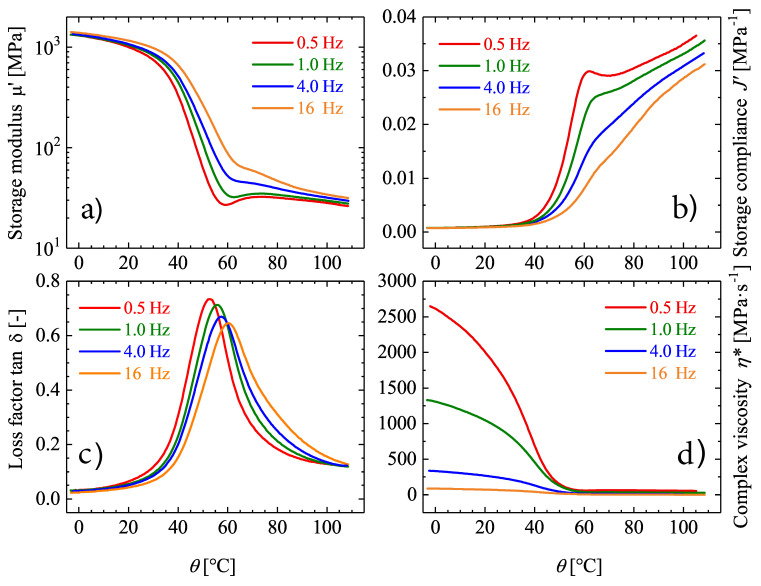
(**a**–**d**): Comprehensive thermo-rheological characterization of Estane during temperature sweep test from 50 to 110 ${}^{\circ }$C with different frequencies ranging from 0.5 to 16 Hz.

In order to study the thermo-rheological properties of Estane, temperature sweep tests and frequency sweep tests have to be conducted. The storage- ($\mu^{\prime }$) and loss shear moduli ($\mu^{\prime \prime }$) of Estane are determined by temperature sweep tests at constant frequencies of: 0.5, 1, 4, 16 Hz with a strain amplitude of $\gamma_{0}=0.01\%$ over a specified temperature domain. Complementary, helpful data of the complex viscosity $(\eta^{*}$) can also be computed in torsion-controlled mode as depicted in Figure 4d. These measurements are specially informative and important in the study of viscoelastic behavior of SMPs and a determinant part of the technique for establishing relaxation transitions. Thereon, precious insights concerning the morphology and structure of the material can be derived.

The results of temperature sweep tests are illustrated in Figure 4a. Here, the temperature sweep tests have been conducted for different frequencies ranging from 0.5 to 16 Hz. It is clear that the storage modulus $\mu^{\prime }$ of Estane decreases gradually with temperature. This decrease of $\mu^{\prime }$ with respect to temperature has a Boltzmann form, typical for physically cross-linked materials [54] as the investigated polymer. The inflection point of the function can be interpreted as $\theta_{g}$. It can be observed that an increase in applied frequency shifts the inflection point of the $\mu^{\prime }-\theta$ curve to higher temperatures. This means a broad transition range of the sample. However, the reduction of storage modulus ($\mu^{\prime }$) at higher frequencies is less pronounced. Another characteristic temperature which can be observed in these graphs is located at temperatures higher than $\theta_{g}$ and is only distinctly observable in tests with lower frequencies. This temperature is assigned for another relaxation inside the polymer chains due to molecular heterogeneities of the polymer. Samples subjected to higher frequencies do not have enough time to represent this relaxation.

In addition to storage modulus, the storage compliance $J^{\prime }$ as a function of temperature for different frequencies have been demonstrated for Estane in Figure 4b. The progress of storage compliance $J^{\prime }$ with temperature can be considered as the mirrored pathway of storage modulus with different scales. The storage compliance $J^{\prime }$ increases gradually with respect to temperature until it reaches its maximum and then changes linearly.

As illustrated in Figure 4c, the $\theta_{g}$ range can be determined from the plot of the loss factor $\tan\delta =\mu^{\prime \prime }/\mu^{\prime }$ versus temperature for different frequencies. Here, three spans can be recognized out of two plateau regions in two temperature ranges from 0 to 40 ${}^{\circ }$C and from 60 to 105 ${}^{\circ }$C with an obvious maximum at near 55 ${}^{\circ }$C for ω=1 Hz. This peak can be assigned to the $\theta_{g}$ of the existing hard and soft domains. As can be observed, the peak values and the corresponding temperatures are also properly recognizable for all other frequencies. However, a little decrease in the maximum and a small shift towards higher temperatures for tanδ can be seen for higher frequencies. Physical interpretability of the loss factor is given here due to the observation that in contrast to the glassy state plateau, and the rubbery state plateau is characterized by higher energy dissipation [54].

Additionally, in torsion-controlled experiments, where a defined torque is applied, and the resulting strain is measured, the complex shear compliance ($J^{*}$) is quantified. $J^{*}$ is the inverse of reciprocal of the complex shear modulus and describes the strain retardation, since the sample needs a defined duration, a so-called retardation time, to migrate from the original configuration to the desired strain, which happens after applying the stress.

#### 3.1.2. Thermal Expansion Experiments

**Figure 5:**
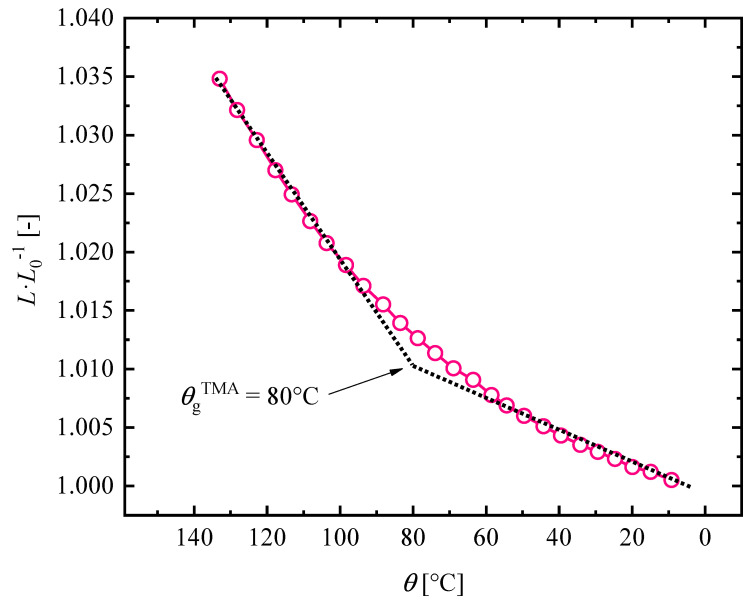
Evolution of thermal strains of Estane samples during cooling from 120 to 0 ${}^{\circ }$C. A piece-wise linear function is fitted to the recorded thermal expansion data. The results are averaged out of three test runs.

With TMA, one can determine the amount of thermal strains. This is also important for modeling purposes especially when the structural relxations for glass forming polymers should be incorporated. Besides DMTA analysis, the coefficients of thermal expansion can be calculated below and above $\theta_{g}$. Figure 5 illustrates the relative changes in specimen length relative to initial length after heating with respect to inverse temperature. In addition to this, in the offset of Figure 5, the results of thermal expansion experiments for the temperature range of 5 to 140 ${}^{\circ }$C are depicted. Our experimental results show that the thermal strains of Estane diminish as the temperature decreases. The steepness of thermal coefficient changes at the point of $\theta_{g}$, which builds up a kink shape in the middle of the graph. Below $\theta_{g}$, a thermal coefficient of 1.27 × $10^{-6}$ ${\;}^{\circ }$C and above $\theta_{g}$, a thermal coefficient of 4.86 × $10^{-6}$ ${\;}^{\circ }$C have been discovered. The obtained $\theta_{g}$ of the CTE experiment is distinctly higher than the $\theta_{g}$ of DMTA experiment. A similar founding was also reported by Westbrook and Qi over acrylate-based polymer networks [55]. It should be noted that, since determination of $\theta_{g}$ is very method-dependent, a one-to-one comparison is not possible.

#### 3.1.3. Torsional Stress Relaxation Experiments

In analogy to large strain uni-axial tensions, torsion experiments give rise to large isochoric deformations. Even at low or moderate strains, such experiments involve inelastic distortions, which are auspicious for thermoplastic elastomers with very limited elastic regions [56]. Therefore, before any simulational studies and for an appropriate characterization of our polymers system stress relaxation experiments were carried out in torsion mode in addition to DMTA experiments. Here, the samples were twisted and kept constrained at different twist angles, whereby their relaxation profiles were obtained as torque-time (M−t) curves. Such stress relaxation experiments were explicitly performed for different twist angles as follows: φ = 45, 90, 360 and 450${}^{\circ }$, at distinct temperatures of 65, 80, 90, 105 ${}^{\circ }$C, as reported in Section 2.3.

**Figure 6:**
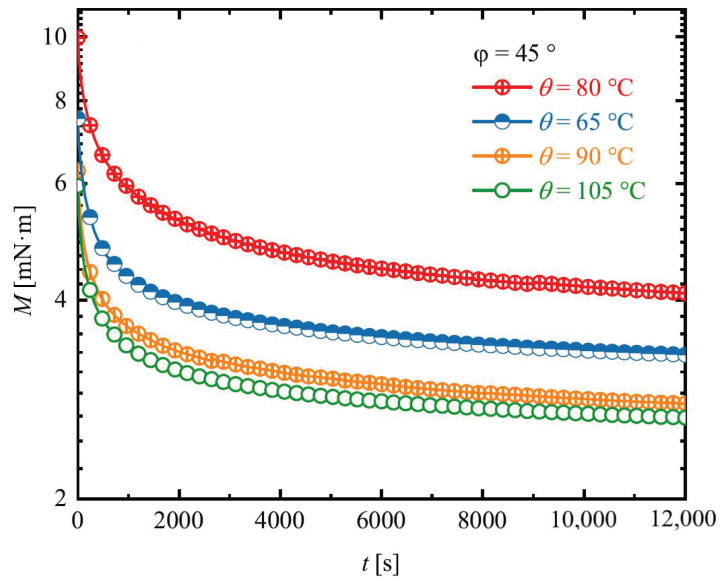
Stress relaxtion experiments for Estane deformed up to φ = 45${}^{\circ }$ at different temperatures as torque-time (M−t) graph.

Since the qualitative stress relaxation responses of the samples for different deflection angles are similar, in Figure 6, the characteristic stress relaxation curves for Estane deformed up to φ = 45${}^{\circ }$ are only depicted at different programming temperatures. In Figure 6, the time t=0 corresponds to the start of the stress relaxation. At a twisting rate of $\dot{\varphi }$ = 3.6${}^{\circ }$/s, the samples have been twisted to φ = 45${}^{\circ }$ and the φ-value was kept constant for 4 h. Here, the influence of temperature on mechanical properties of the samples is noticeable. With an increase in the programming temperature, polymer samples become more flexible and less torque is needed for deformation. This is a consequence of an increase in the kinetics energy of every single polymer chains, which results in an increase in existing free volume. From the results illustrated in Figure 6, one can conclude that, during the experimental time, none of the samples relax to zero, and a small amount of stresses remain in polymers, that relax with much slower time.

**Figure 7:**
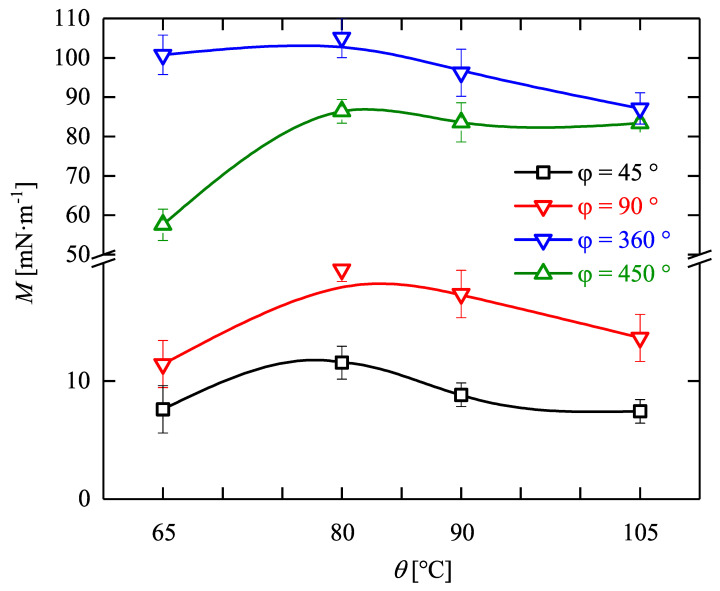
Evolution of maximum torque needed to deform Estane up to different twist angles at distinct programming temperatures.

Intriguingly, at θ = 80${}^{\circ }$, the needed torque for torsion is higher than that for θ = 65${}^{\circ }$. This would be a hint for a kind of thermal stiffening which needs further experimental investigations. This has also been observed for all other deflection angles as depicted in Figure 7.

In this picture, the maximum amount of the torques needed to twist the samples up to different deflection angles and temperatures are illustrated. It should be mentioned that, as expected, the maximum amount of torque needed to twist the samples increase with an increase in the amount of deflection angle. However, for a torsion of φ = 450${}^{\circ }$, the torque needed for deformation of the samples reduces, and about 10% less torque is needed in comparison to φ = 360${}^{\circ }$ at a temperature of θ = 80 ${}^{\circ }$C. It is assumed that during large amount of entropic and less amount of enthalpic deformation processes, some intermolecular forces, like London dispersion forces and H-bonds, which strongly depend on atomic distances, are broken during a plastic deformation.

**Figure 8:**
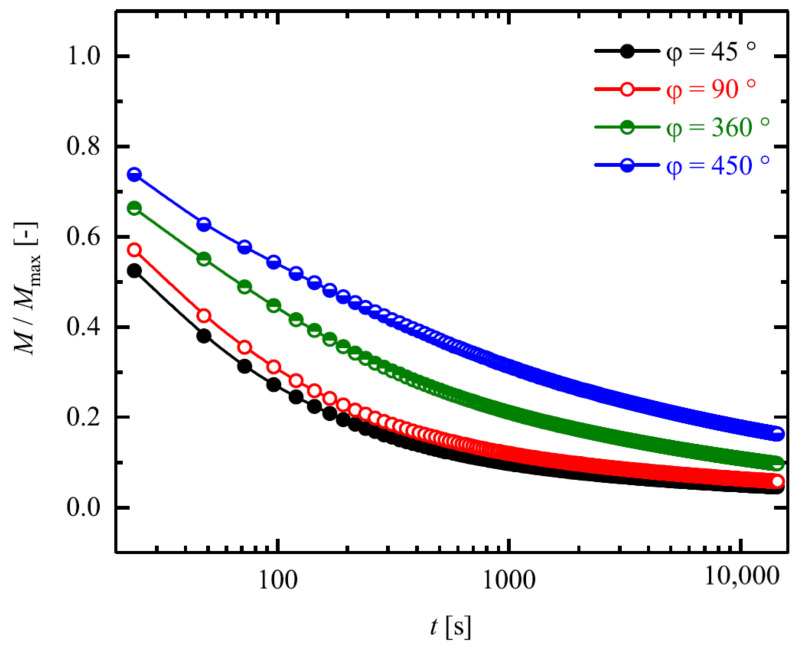
Evolution of the torque in relative units to deform the samples up to different deflection angles during relaxation at $\theta_{\mathrm{prog}}$${}^{\circ }$C.

Finally, in Figure 8, the kinetics of stress relaxations of different deflection angles are depicted in semi-logarithmic form in relative units. According to the results represented in Figure 8, samples twisted to lower deflection angles not only are relaxed faster but also much higher amount of the torques is recovered. As the samples deformed up to φ = 45${}^{\circ }$ recover more than 50% of their torque in only 100 s, samples twisted to φ = 450${}^{\circ }$ recover only 40% of their torque.

### 3.2. Identification of Material Parameters

#### 3.2.1. Assumption of Thermo-Rheological Simplicity

Since DMTA experiments can only be reported for a limited range of frequency and temperature domain, they are insufficient for a complete description of polymer’s viscoelasticity. In order to overcome this problem and to follow the long term viscoelastic behavior of polymers, for thermo-rheological simple materials, the TTSP is exerted. To apply the TTSP, one should perform frequency sweep tests at different temperatures. Then, the storage and loss moduli for one temperature is chosen as reference, and other results are shifted to right or left to obtain continuous mastercurves

(17) $$\begin{array}{c} \begin{array}{c} \mu^{\prime }\left(a_{T}^{j}\;\omega ,\theta \right)=\mu^{\prime }\left(\omega ,\theta_{\mathrm{ref}}\right),\\ \mu^{\prime \prime }\left(a_{T}^{j}\;\omega ,\theta \right)=\mu^{\prime \prime }\left(\omega ,\theta_{\mathrm{ref}}\right). \end{array} \end{array}$$

**Figure 9:**
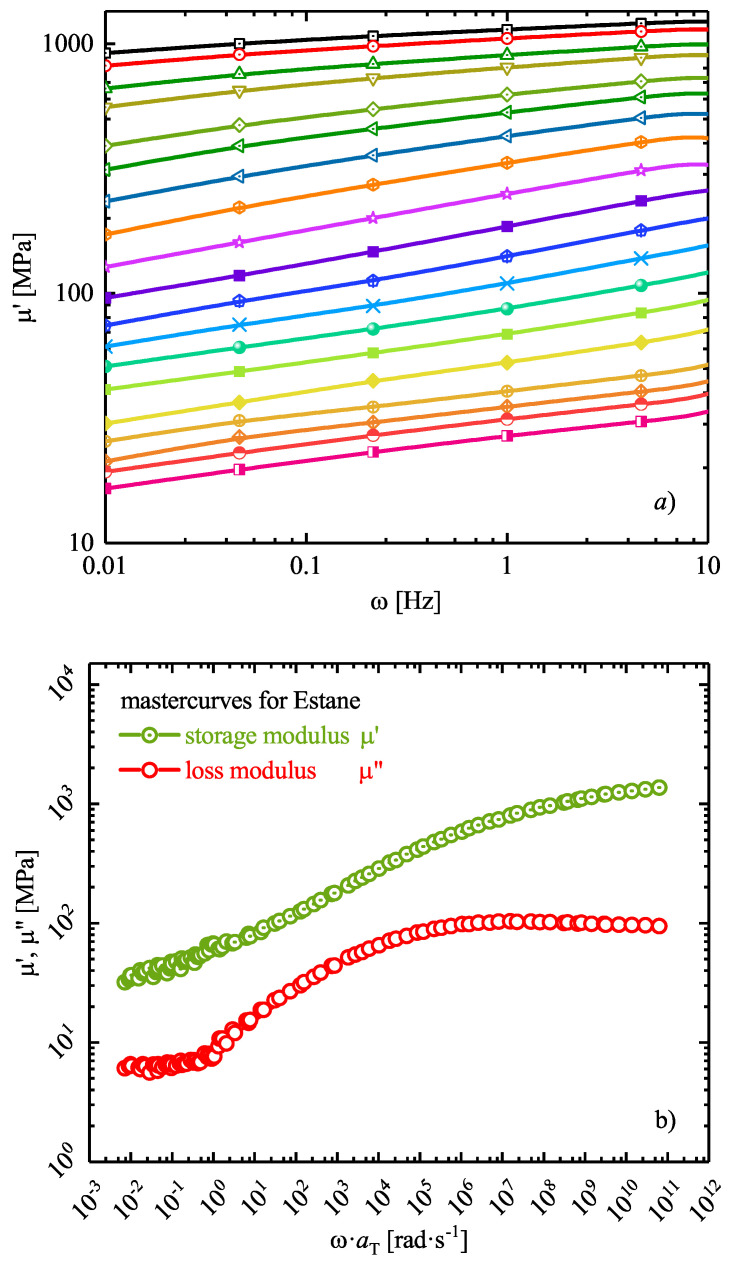
(**a**) Storage modulus as a function of frequency at different isothermal conditions obtained from Dynamic Mechanical Thermal Analysis (DMTA) experiments and (**b**) mastercurves of storage ($\mu^{\prime }$) and loss moduli ($\mu^{\prime \prime }$) shifted to a reference temperature of $\theta_{\mathrm{ref}}=50^{\circ }$. The solid line in the insert show the calculated shift factors obtained from WLF approximation.

Here, $\theta_{\mathrm{ref}}$ and $a_{T}^{j}$ denote the reference temperature and the horizontal shift factor, respectively. Horizontal shift factor is linked to the chosen $\theta_{\mathrm{ref}}$ according to WLF equation

(18) $$a_{T}^{j}\left(\theta \right)=\exp\left\{-\frac{C_{1}\left(\theta -\theta_{\mathrm{ref}}\right)}{C_{2}+\theta -\theta_{\mathrm{ref}}}\right\},$$

where $C_{1}$ and $C_{2}$ are material parameters. In Figure 9a, the measured storage moduli of Estane at different temperatures ranging over [5–100] ${}^{\circ }$C (in steps of every 5 ${}^{\circ }$C) are depicted in the range of unreduced angular frequencies [$10^{-2}$–$10^{2}$] rad s${}^{-1}$. With these data and resulted loss moduli (not shown here), the corresponding mastercurves of $\mu^{\prime }$ and $\mu^{\prime \prime }$ shifted to $\theta_{\mathrm{ref}}$ = 50 ${}^{\circ }$C are achieved and depicted in Figure 9b. For data shown in this picture, $C_{1}$ and $C_{2}$ have been identified and listed in Table 1, together with calculated storage- and bulk modulus for Estane.

As can be seen from Figure 9b, the chosen temperature increment is sufficient so that with the material parameters derived from WLF equation, a particularly fine continuous mastercurve could be obtained.

Now, the time-dependent behavior of thermo-viscoelastic model can be described by fitting the right material parameters. Since polymer relaxation occurs with a spectrum of relaxations, normally a generalized Maxwell-Zener model is utilized, as a suitable constitutive framework for thermo-rheologically simple polymers with *n* superimposed relaxation processes. To find the true material parameters, two procedures are generally applied if and only if the system is thermo-rheologically simple. The first one is based on linearizion of finite-strain model to construct a corresponding small-strain viscoelastic model with a continuous relaxation spectrum as proposed by Haupt et al. [57] and further developed by others [58]. The second approach is based on determination of Maxwell-model parameters according to obtained experimental DMTA experiments and resulted mastercurve [56].

Here, in an attempt to accurately represent the continuous viscoelastic spectrum as measured using DMTA, the second approach is employed. To do so, n=19 discrete nonequilibrium Maxwell elements are taken into account and the constitutive behavior of the Maxwell element is defined by Prony series coefficients, optimized by the Tikhonov regularization method [59]

(19) $$\mu^{\prime }\left(\omega ,\theta \right)=\mu_{\mathrm{eq}}+\sum_{m=1}^{N}\mu_{m}^{j}\frac{\omega^{2}\tau_{m,j}^{2}\left(\theta \right)}{1+\omega^{2}\tau_{m,j}^{2}\left(\theta \right)},$$

(20) $$\mu^{\prime \prime }\left(\omega ,\theta \right)=\sum_{m=1}^{N}\mu_{m}^{j}\frac{\omega \tau_{m,j}\left(\theta \right)}{1+\omega^{2}\tau_{m,j}^{2}\left(\theta \right)}.$$

The resulted storage- and loss modulus mastercurves for the reference temperature $\theta_{\mathrm{ref}}=50{\;}^{\circ }$C are shown in Figure 9b, and a suitable series of relaxation times $\tau_{m,j}$ and relaxation moduli $\mu_{m}^{j}$ are defined

(21) $$\mu \left(t\right)=\mu_{\mathrm{eq}}+\sum_{m=1}^{N}\mu_{m}^{j}\left(\theta \right)\cdot \exp\left(-\frac{t}{\tau_{m,j}\left(\theta \right)}\right).$$

Here, $\mu_{\mathrm{eq}}$, $\mu_{m}$, and $\tau_{m}\left(\theta \right)$ are the equilibrium modulus, the overall modulus of the *j*th Maxwell unit, and its relaxation times, respectively. The best fitting Prony coefficients are listed in Table 2. It should be noted that the elastic shear modulus of the neo-Hookean equilibrium element is evaluated in the limit $\lim_{\;\omega \to 0}\mu^{\prime }\left(a_{T}^{j}\;\omega \right)$ as $\mu_{\mathrm{eq}}$ = 7.020. However, the absolute value is not decisive for most modeling purposes, since the material response is dominated by viscous stresses.

#### 3.2.2. Assumption of Thermo-Rheological Complexity

Another strategical procedure applied to identify the material parameters is supported by the assumption of thermo-rheological complexity of Estane. The thermo-rheological complexity, in contrast to thermo-rheological simplicity means that all relaxation times are influenced by temperature in a different way. This deliberated methodology is based on four steps. First, the introduced material model is adopted to the mechanical behavior of Estane at the lowest temperature. This is because the relaxation behavior at this temperature is highly pronounced. It should be noted that the relaxation tests have been performed in the range of [10–80] ${}^{\circ }$C and must not be exceeded in the modeling experiments. Second, it is supposed that the shear moduli of the Maxwell branches depend on temperature in the same way as the relaxation times do. This means

(22) $$\tau_{i}^{j}\left(\theta \right)=\tau_{0}^{j}\;a^{j}\left(\theta \right)\;\;\;\;\;\mathrm{and}\;\;\;\;\;\mu_{i}^{j}\left(\theta \right)=\mu_{0}^{j}\;a^{j}\left(\theta \right).$$

Third, the constitutive Equations (12)–(14) are fitted to the experimental relaxation curve at θ = 10 ${}^{\circ }$C and so are Prony series coefficients determined. For this purpose, first the number of dissipative branches has to be estimated and then for each Maxwell unit, its own shift factor $a^{j}\left(\theta \right)$ must be specified. It is well documented that up to two Maxwell units per time decade are required to adequately represent the viscoelastic behavior of the material. Here, six Maxwell branches have been chosen. The material parameters are identified by Levenberg-Marquardt algorithm and are listed in Table 3.

**Figure 10:**
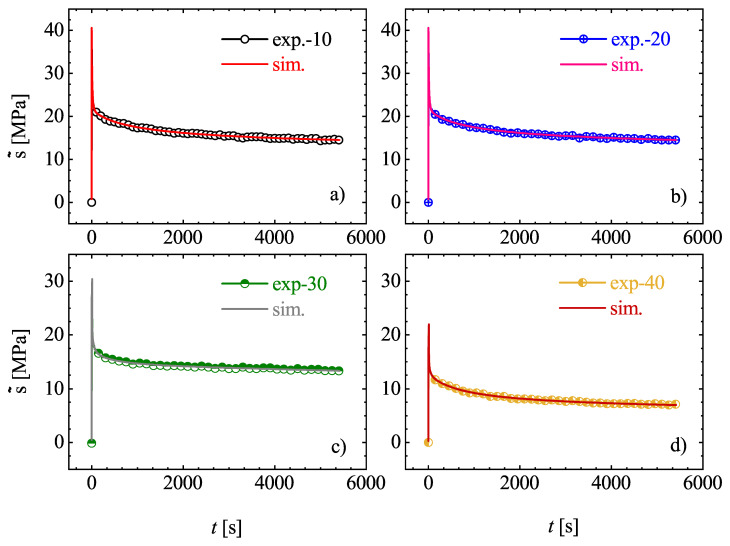
(**a**–**d**): Comparison of computational and experimental stress relaxation tests with different isothermal temperatures under $\theta_{g}$.

**Figure 11:**
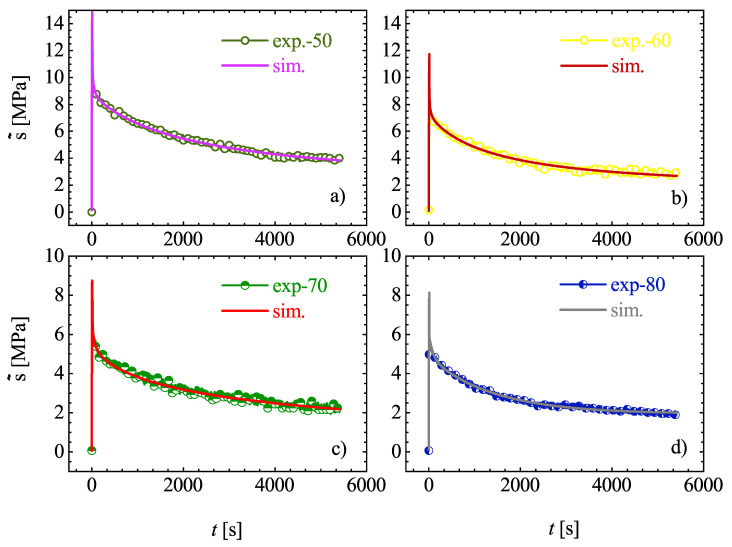
(**a**–**d**): Comparison of computational and experimental stress relaxation tests with different isothermal temperatures above $\theta_{g}$.

**Figure 12:**
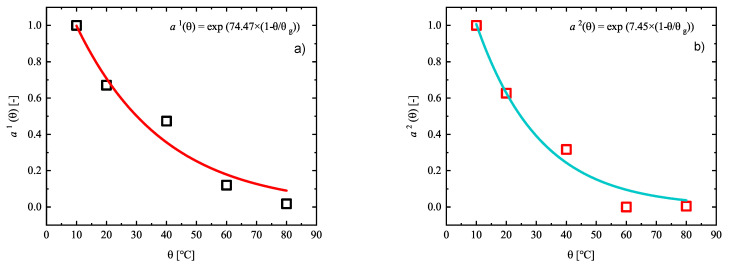

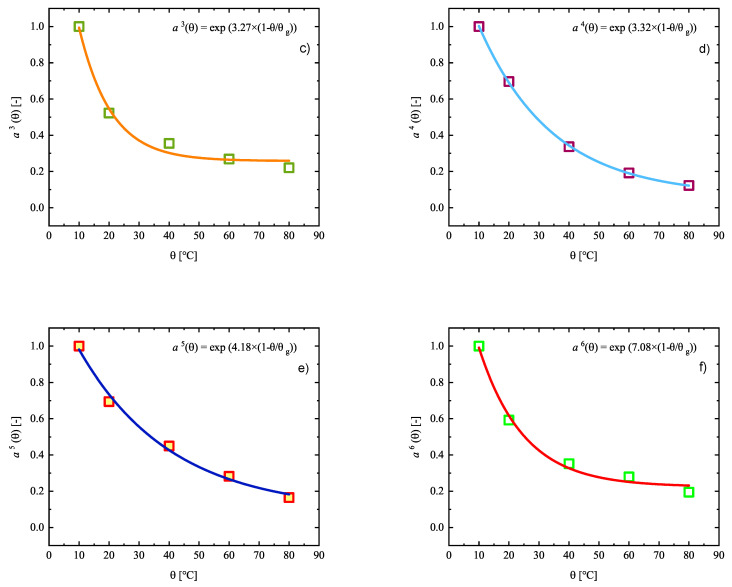
(**a**–**f**): Identification of the shift functions $a^{j}\left(\theta \right)$ for the set of six Maxwell branches.

Finally, the identified relaxation times $\tau_{m,j}$ of the Maxwell elements are accepted as constants for all other temperatures. To determine the shift functions ($a^{j}\left(\theta \right)$), the identified shear moduli at a temperature of 80 ${}^{\circ }$C is now assumed to be the upper bound and the shear moduli $\mu^{j}\left(\theta \right)$ at the other temperatures are progressively determined by fitting the modeling results to experiments. Figure 10 and Figure 11 show the simulations and experiments using the determined parameters $\mu^{j}\left(\theta \right)$ with constant relaxation times $\tau_{m,j}$. In order to determine the shift functions $a^{j}\left(\theta \right)$, the identified material parameters $\mu^{j}\left(\theta \right)$ are plotted over the absolute temperature θ. A standardization with respect to the initial values $\mu^{j}\left(\theta \right)$ permits the conclusion that material can be modeled by choosing a separate function $a^{j}\left(\theta \right)$ for each Maxwell element. Although, it is also quite suitable to set the same function $a^{j}\left(\theta \right)\;=\;a^{j}\left(\theta \right)$ for all Maxwell elements. Concerning the temperature of 10 ${}^{\circ }$C, the shift function has to be equal to 1, and, for higher temperatures, it has to decrease so that, for $\mu^{j}\left(\theta \right)$, a value near zero can be reached. This circumstances are illustrated best over an exponential approach, which is reminiscent of the Arrhenius equation [32]. In the illustrations of Figure 12, the exponential approaches are represented for the 6 Maxwell elements due to the data from Figure 10 and Figure 11.

### 3.3. Validation of the Model

**Figure 13:**
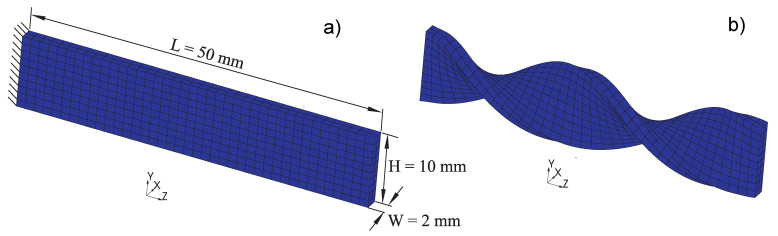
(**a**): Geometry of the beam sample and the Finite Element Method (FEM) mesh in its initial (undeformed) configuration and (**b**): The shape of the sample twisted up to 360${}^{\circ }$ at t=300 s.

In order to validate the finite strain material model, two torsion tests are considered here. The experiments are carried out at different temperatures and involve finite strains. A rectangular beam sample with dimensions L × W × H = 50 mm × 2 mm × 5 mm is clamped at one end as shown in the left hand side of Figure 13. The opposite end is assumed to be rigid. The elongation of the beam is not restrained. The twist of the beam is explicitly prescribed as a function of time. Within the first 300 s, the twist angle increases linearly from 0 to 2π rad, after that the twist is held constant.

**Figure 14:**
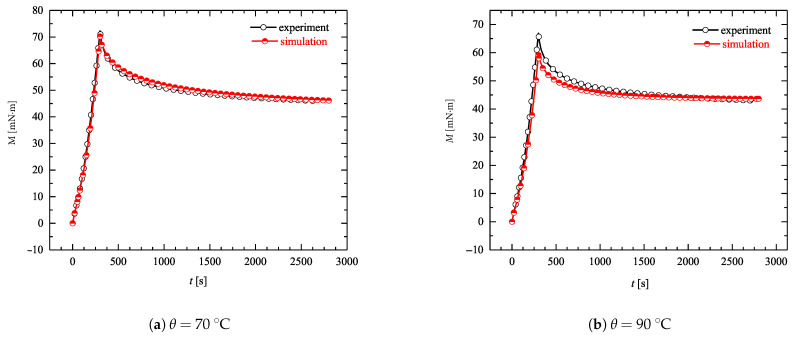
Experimental and simulated torque-time (M−t) graphs for torsion tests at two different programming temperatures $\theta =70\;{}^{\circ }$C and $\theta =90\;{}^{\circ }$C.

The torsion tests are numerically simulated with the Finite Element Method (FEM) using the software package MSC.MARC. Toward that end, an FEM mesh containing 800 elements of class hex20 with a quadratic interpolation is utilized (see Figure 13 (left)). The required Prony series coefficients are obtained from the assumption of thermo- rheological simplicity. Simulations are performed for two different temperatures; WLF coefficients are taken from Table 1. The experimental data and corresponding FEM results are plotted in Figure 14. In this figure, the relaxation stage which follows the active loading is clearly visible. As can be seen, the assumption of the thermo-rheological simplicity enables sufficiently accurate simulations even in the essentially heterogeneous case of beam torsion. Since the temperature interval is relatively small, the identified set of parameters provides a very good accuracy in the considered range of strain rates.

## 4. Discussion and Conclusions

Apart from the accuracy and flexibility, the geometrically nonlinear approach of Simo and Miehe is highly practical. Owing to the explicit update Equation (16), the time stepping is robust and efficient. This computational efficiency becomes important when dealing with large-scale FEM simulations: Depending on the number of finite elements, stress integration points per each element, time steps, and the average number of iterations, the number of calls of the material subroutine ranges up to $10^{10}$. If the model contains 20 Maxwell branches, this means $2\times 10^{11}$ calls of the Maxwell material’s subroutine. Another advantage of the implemented non-iterational time-stepping method is the exact preservation of the incompressibility condition. For such algorithms, the accumulation of the numerical error is suppressed even when working with large time steps and strain increments [48].

From the theoretical standpoint, the model is thermodynamically consistent, objective and free from spurious shear oscillations. The stress response exhibits a pure split into volumetric and deviatoric parts [37], which is useful when modeling incompressbile or nearly incompressible materials. Interestingly, both thermo-rheological simple and complex approaches yield similar numerical schemes. The main difference lies in calibration and validation of the modeling assumptions, as well as their applicability domains.

A relatively large number of rheological Maxwell units is implemented to represent the relaxation behavior over a broad range of temperatures and strain rates. To solve this problem, fractional time rates are usually implemented [2], at a cost of more complex numerical methods. However, a simpler way to reduce the number of Maxwell branches is to incorporate stress-dependent viscosities [1].

The main conclusion of this study is that in a certain range of temperature, strain, and strain rate Estane behaves as a thermo-rheological simple material, which can be described by a generalized finite strain Maxwell-Zener model.

When modeling Estane as a thermo-rheological complex medium, a pragmatic approach is advocated, assuming temperature-dependent shear moduli; see Equation (22). This temperature-dependence can occasionaly violate the 2nd law of thermodyamics. Therefore, the model should be restricted to a specific range of strains and temperatures. The development of an accurate material model which a priori satisfies the 2nd law of thermodynamics still remains an open problem. A promising line of research is to incorporate a chemical potential $\psi_{\mathrm{chem}}$ into the additive decomposition of the free energy (9). Thus, the evolution of the stiffness (Equation (22)) can be brought into compliance with the energy balance. 

## Figures and Tables

**Table 1:** Williams-Landel-Ferry (WLF) constants for Estane.

$\mu_{\mathrm{eq}}$ [MPa]	*k* [MPa]	$C_{1}$	$C_{2}$	$\theta_{\mathrm{ref}}$ [°C]
7.020	700	8	18	50

**Table 2:** Generalized Maxwell model relaxation times $\tau_{m,j}$ and associated shear moduli $\mu_{m}^{j}$ considering thermo-rheological simplicity for Estane.

Material Parameter	1	2	3	4	5
$\mu^{j}$ [MPa]	8.495 × 10${}^{1}$	7.954 × 10${}^{1}$	7.414 × 10${}^{1}$	6.874 × 10${}^{1}$	6.334 × 10${}^{1}$
$\tau^{j}$ [s]	1.000 × 10${}^{-9}$	5.995 × 10${}^{-9}$	3.594 × 10${}^{-8}$	2.154 × 10${}^{-7}$	1.291×10${}^{-6}$
Material Parameter	6	7	8	9	10
$\mu^{j}$ [MPa]	5.7947 × 10${}^{1}$	5.254 × 10${}^{1}$	4.713 × 10${}^{1}$	4.168 × 10${}^{1}$	3.602 × 10${}^{1}$
$\tau^{j}$ [s]	7.743 × 10${}^{-6}$	4.641 × 10${}^{-5}$	2.782 × 10${}^{-4}$	1.668 × 10${}^{-3}$	1.000 ×10${}^{-2}$
Material Parameter	11	12	13	14	15
$\mu^{j}$ [MPa]	2.932 × 10${}^{1}$	2.106 × 10${}^{1}$	1.399 × 10${}^{1}$	9.998 × 10${}^{0}$	8.412 × 10${}^{0}$
$\tau^{j}$ [s]	5.995 × 10${}^{-2}$	3.594 × 10${}^{-1}$	2.154 × 10${}^{0}$	1.291 × 10${}^{1}$	7.743 × 10${}^{1}$
Material Parameter	16	17	18	19	
$\mu^{j}$ [MPa]	7.613 × 10${}^{0}$	6.331 × 10${}^{0}$	4.651 × 10${}^{0}$	2.823 × 10${}^{0}$	
$\tau^{j}$ [s]	4.641 × 10${}^{2}$	2.782 × 10${}^{3}$	1.668 × 10${}^{4}$	1.000 × 10${}^{5}$	

**Table 3:** Identified material parameters based on finite strain relaxation tests at θ = 10 ${}^{\circ }$C.

Material Parameter	1	2	3	4	5	6
$\mu^{j}$ [MPa]	74.47	7.45	3.27	3.32	4.18	7.08
$\tau^{j}$ [s]	130.13	4.91	849.46	604.72	2,297,500	3,617,300

## Data Availability

The data presented in this study are available on request from the corresponding author.
